# Genomic insights into the thiamin metabolism of *Paenibacillus thiaminolyticus* NRRL B-4156 and *P. apiarius* NRRL B-23460

**DOI:** 10.1186/s40793-017-0276-9

**Published:** 2017-10-03

**Authors:** David Sannino, Esther R. Angert

**Affiliations:** Cornell University, Ithaca, NY USA

**Keywords:** Thiaminase I, Paenibacillus thiaminolyticus, Paenibacillus apiarius, Paenibacillus dendritiformis, Thiamin, Hydroxymethyl pyrimidine

## Abstract

*Paenibacillus thiaminolyticus* is the model organism for studying thiaminase I, an enigmatic extracellular enzyme. Originally isolated from the feces of clinical patients suffering from thiamin deficiency, *P. thiaminolyticus* has been implicated in thiamin deficiencies in humans and other animals due to its ability to produce this thiamin-degrading enzyme. Its close relative, *P. apiarius,* also produces thiaminase I and was originally isolated from dead honeybee larvae, though it has not been reported to be a honeybee pathogen. We generated draft genomes of the type strains of both species, *P. thiaminolyticus* NRRL B-4156 and *P. apiarius* NRRL B-23460, to deeply explore potential routes of thiamin metabolism. We discovered that the thiaminase I gene is located in a highly conserved operon with thiamin biosynthesis and salvage genes, as well as genes involved in the biosynthesis of the antibiotic bacimethrin. Based on metabolic pathway predictions, *P. apiarius* NRRL B-23460 has the genomic capacity to synthesize thiamin *de novo* using a pathway that is rarely seen in bacteria, but *P. thiaminolyticus* NRRL B-4156 is a thiamin auxotroph. Both genomes encode importers for thiamin and the pyrimidine moiety of thiamin, as well as enzymes to synthesize thiamin from pyrimidine and thiazole.

## Introduction

Prior to World War II, beriberi and other vitamin deficiencies were prevalent in Japan and linked to a diet composed almost entirely of polished rice [1]. Additionally, it was discovered that certain fish and shellfish contained no thiamin and moreover any thiamin added to these raw foodstuffs was quickly destroyed [2]. While investigating potential links between the intestinal microbiota and beriberi, Shibata and colleagues found that when thiamin was added to feces or infused in the colon of patients suffering thiamin deficiency, the added thiamin disappeared [2, 3]. The thiaminase enzyme responsible for the destruction of thiamin in feces and in animal tissues was discovered shortly thereafter. Several bacteria, including 10.1601/nm.5156 *,* were isolated by Matsukawa and Misawa from patient fecal samples with thiaminase activity [2]. The discovery of thiaminase producing bacteria facilitated extensive research efforts to understand the biochemistry of thiaminase and the biology of 10.1601/nm.5156 [4].

10.1601/nm.5156 became a model system for studying the secreted bacterial thiaminase now known as thiaminase I [5–10]. Thiaminase I catalyzes the base substitution of the thiazole moiety of thiamin with numerous organic nucleophiles such as pyridine, quinolone, or compounds containing a sulfhydryl group, like cysteine [2, 10, 11]. Early studies of this extracellular enzyme found that thiaminase I activity is repressed when high concentrations of thiamin are added to cultures and culture supernatant [8, 9]. The crystal structure of 10.1601/nm.5156 thiaminase I revealed that the 42 kDa protein has a catalytic cysteine residue and the protein is structurally similar to the group II periplasmic binding proteins, particularly the thiamin-binding protein TbpA in *E. coli* [12]. We recently found that 10.1601/nm.5116 also has thiaminase I activity (unpublished). This close relative of 10.1601/nm.5156 was originally isolated from the larvae of dead honeybees, although it was not the causative agent of their death [13]. Despite the extensive biochemical and mechanistic understanding of the enzyme, the biological function and context in which 10.1601/nm.5116, 10.1601/nm.5156 and other thiaminase I producers use thiaminase I remains a mystery [14].

Although thiaminase I activity is found in plants such as bracken fern [15] and nardoo [16], as well as in animals such as crustaceans, ruminants, and fish, the only confirmed producers of thiaminase I are microbial, including one eukaryote, the amoeba *Naegleria gruberi* [15, 17, 18]. Thiaminase I activity in food contributes to thiamin deficiency in animals and is implicated in Early Mortality Syndrome in salmonids in the Great Lakes and Baltic Sea [18]. A link between 10.1601/nm.5156 and this thiamin deficiency syndrome has been suggested, as 10.1601/nm.5156 has been isolated from the viscera of alewife, a fish with high thiaminase activity that is a food source for Great Lakes salmonids. Additionally, it was demonstrated that Early Mortality Syndrome could be induced in lake trout fed an experimental diet supplemented with 10.1601/nm.5156 [18, 19]. As with humans, 10.1601/nm.5156 is not always isolated from intestinal contents of fish with high thiaminase I activity so other sources of the enzyme likely impact thiamin metabolism in populations of animals [20].

Thiaminase I enzymes are not widely distributed in the microbial world and are produced by a small subset of phylogenetically diverse microorganisms. By sequencing the genomes of the type strains, 10.1601/nm.5156 10.1601/strainfinder?urlappend=%3Fid%3DNRRL+B-4156 and its relative 10.1601/nm.5116 10.1601/strainfinder?urlappend=%3Fid%3DNRRL+B-23460, we aim to establish the genomic context of the thiaminase I gene to help gain a better understanding of the biological function of the enzyme. The draft genomes have helped uncover the routes of vitamin B1 metabolism available to these bacteria, which will help inform our model of the ecological role of thiaminase I, and perhaps its contribution to vitamin deficiencies in animals.

## Organism information

### Classification and features

The original isolate of 10.1601/nm.5156 *,* classified as 10.1601/nm.4999 *,* was obtained from the feces of a Japanese patient suffering from thiamin deficiency and chronic constipation [2]. Additional strains of 10.1601/nm.5156 have been isolated from fecal samples of healthy human subjects from Kyoto and Ube City, as well as those with symptoms of thiamin deficiency [2, 4]. Aside from being associated with human feces, 10.1601/nm.5156 reportedly induced bacteremia in an 80-year-old hospital patient undergoing hemodialysis for end-stage renal disease [21]. Strains of 10.1601/nm.5156 have been found in the alimentary tract and feces of thiamin deficient lambs, ewes, and sheep [22], and from the viscera of Lake Michigan alewives [18, 23]. Other isolates have been recovered from honeybees [24] and from soil [4]. Growth of 10.1601/nm.5156 on defined minimal media requires the addition of thiamin or the two moieties that form thiamin [6]. Like some strains of 10.1601/nm.5156, 10.1601/nm.5116 was isolated from dead honeybee larvae, adults, and honeycombs [13]. It is not suspected to be a honeybee pathogen as 10.1601/nm.5116 spores fed to larvae and adults did not induce death or any obvious pathology [13]. A few 10.1601/nm.5156 strains have been erroneously classified as 10.1601/nm.5116 [25]. In contrast to 10.1601/nm.5156, 10.1601/nm.5116 has not been studied extensively.

**Fig. 1:**
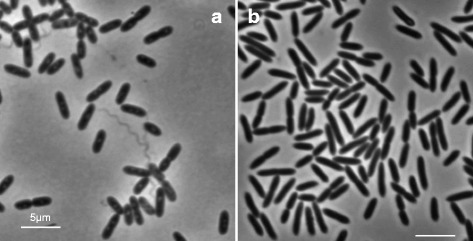
Phase-contrast micrographs of 10.1601/nm.5116 10.1601/strainfinder?urlappend=%3Fid%3DNRRL+B-23460 and 10.1601/nm.5156 10.1601/strainfinder?urlappend=%3Fid%3DNRRL+B-4156. **a** Depicts 10.1601/nm.5116 10.1601/strainfinder?urlappend=%3Fid%3DNRRL+B-23460 cells grown in 10.1601/strainfinder?urlappend=%3Fid%3DTSB+for+24 hr. at 30 °C. **b** Depicts 10.1601/nm.5156 10.1601/strainfinder?urlappend=%3Fid%3DNRRL+B-4156 cells grown in 10.1601/strainfinder?urlappend=%3Fid%3DTSB+for+24 hr. at 37 °C. Scale bars represent 5 μm

Both species are rod-shaped endospore formers and produce a single ellipsoid endospore in a swollen sporangium, with the spore coat of 10.1601/nm.5116 described as unusually thick [25]. The spore produced by 10.1601/nm.5116 has a rectangular outline, unlike the more ellipsoid shape seen in 10.1601/nm.5156 [25]. 10.1601/nm.5116 cells are slightly larger than 10.1601/nm.5156 cells as they range from 3.0–5.0 μm in length and 0.7–0.8 μm in width, while 10.1601/nm.5156 cells are 2.0–3.0 μm long and 0.5–1.0 μm wide [24, 25] (Fig. 1). The predominant cellular fatty acid in both 10.1601/nm.5116 and 10.1601/nm.5156 is anteiso-C_15:0_ [25], and both have a Gram-positive cell wall. General features of the two organisms are summarized in Tables 1 and 2.

**Table 1:** Classification and general features of 10.1601/nm.5116 10.1601/strainfinder?urlappend=%3Fid%3DNRRL+B-23460 [53]

MIGS ID	Property	Term	Evidence code^a^
	Classification	Domain *Bacteria*	TAS [54]
		Phylum 10.1601/nm.3874	TAS [55]
		Class 10.1601/nm.4854	TAS [56, 57]
		Order *Bacilliales*	TAS [58]
		Family *Paenibacilliaceae*	TAS [56]
		Genus 10.1601/nm.5109	TAS [26, 59]
		Species *apiarius*	TAS [25]
		(Type) strain: 10.1601/strainfinder?urlappend=%3Fid%3DNRRL+B-23460 ^*T*^	
	Gram stain	Positive	TAS [25]
	Cell shape	Rod	TAS [25]
	Motility	Motile	TAS [25]
	Sporulation	Endospores with thick coats	TAS [25]
	Temperature range	15–40 °C	TAS [25]
	Optimum temperature	28 °C	TAS [25]
	pH range; Optimum	Not reported	
	Carbon source	D-glucose, D-galactose, cellobiose, maltose, melibiose, sucrose, trehalose, salicin; can hydrolyize starch, casein	TAS [13]
MIGS-6	Habitat	Soil and honeybee associated	TAS [13]
MIGS-6.3	Salinity	5% NaCl (*w*/*v*)	TAS [25]
MIGS-22	Oxygen requirement	facultative	TAS [13]
MIGS-15	Biotic relationship	free-living	TAS [13]
MIGS-14	Pathogenicity	non-pathogen	TAS [25]
MIGS-4	Geographic location	Manitoba, Canada	TAS [13]
MIGS-5	Sample collection	1950s	TAS [13]
MIGS-4.1	Latitude	Not reported	
MIGS-4.2	Longitude	Not reported	
MIGS-4.4	Altitude	Not reported	

^a^ Evidence codes - IDA: Inferred from Direct Assay; TAS: Traceable Author Statement (i.e., a direct report exists in the literature); NAS: Non-traceable Author Statement (i.e., not directly observed for the living, isolated sample, but based on a generally accepted property for the species, or anecdotal evidence). These evidence codes are from the Gene Ontology project [60]

**Table 2:** Classification and general features of 10.1601/nm.5156 10.1601/strainfinder?urlappend=%3Fid%3DNRRL+B-4156 [53]

MIGS ID	Property	Term	Evidence code^a^
	Classification	Domain *Bacteria*	TAS [54]
		Phylum 10.1601/nm.3874	TAS [55]
		Class 10.1601/nm.4854	TAS [56, 57]
		Order *Bacilliales*	TAS [58]
		Family *Paenibacilliaceae*	TAS [56]
		Genus 10.1601/nm.5109	TAS [26, 59]
		Species *thiaminolyticus*	TAS [24]
		(Type) strain: 10.1601/strainfinder?urlappend=%3Fid%3DNRRL+B-4156 ^*T*^	
	Gram stain	Positive	TAS [24]
	Cell shape	Rod	TAS [24]
	Motility	Motile	TAS [24]
	Sporulation	endospores	TAS [24]
	Temperature range	20–45 °C	TAS [24]
	Optimum temperature	28 °C	TAS [24]
	pH range; Optimum	Not reported	
	Carbon source	D-glucose, D-fructose, D-galactose, D-ribose, lactose, cellobiose, maltose, mannose, melibiose, sucrose, trehalose, salicin; can hydrolyze starch, casein	TAS [24]
MIGS-6	Habitat	Soil, animal associated	TAS [24]
MIGS-6.3	Salinity	5% NaCl (w/v)	TAS [24]
MIGS-22	Oxygen requirement	facultative	TAS [24]
MIGS-15	Biotic relationship	free-living	TAS [24]
MIGS-14	Pathogenicity	non-pathogen (1 case in humans)	NAS [21, 24]
MIGS-4	Geographic location	Japan	TAS [24]
MIGS-5	Sample collection	1940s	TAS [24]
MIGS-4.1	Latitude	Not reported	
MIGS-4.2	Longitude	Not reported	
MIGS-4.4	Altitude	Not reported	

^a^ Evidence codes - IDA: Inferred from Direct Assay; TAS: Traceable Author Statement (i.e., a direct report exists in the literature); NAS: Non-traceable Author Statement (i.e., not directly observed for the living, isolated sample, but based on a generally accepted property for the species, or anecdotal evidence). These evidence codes are from the Gene Ontology project [60]

**Fig. 2:**
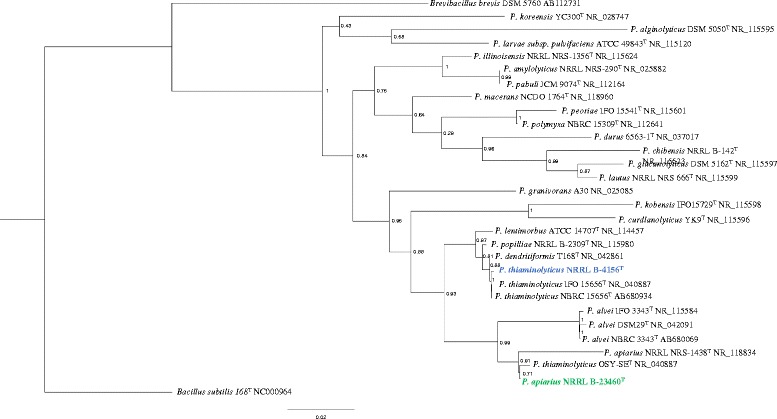
Phylogenetic tree of 10.1601/nm.5109 spp. based on 16S rRNA gene sequences. The maximum likelihood tree was inferred from a comparison of sequences from 10.1601/nm.5109 spp., 10.1601/nm.5168 10.1601/strainfinder?urlappend=%3Fid%3DDSM+5760, and 10.1601/nm.10618 168 using FastTree 2.1 [31]. The sequences generated from the draft genomes of this study are highlighted, with 10.1601/nm.5156 10.1601/strainfinder?urlappend=%3Fid%3DNRRL+B-4156 in blue font, and 10.1601/nm.5116 10.1601/strainfinder?urlappend=%3Fid%3DNRRL+B-23460 in green font

These paenibacilli were originally classified as members of the genus 10.1601/nm.4857, based on their morphological features and biochemical properties, although 10.1601/nm.5116, 10.1601/nm.5156 and their close relatives were not included in the original description of the genus [26]. Due to their similar phenotypes, six strains of 10.1601/nm.4999 were classified in the *B. apiarius* species group, but 16S rRNA gene analysis revealed that *B. apiarius* isolates form two separate clades [25]. This phylogenetic analysis further provided support for reclassifying *B. apiarius* strains as 10.1601/nm.5116 and those clustering with 10.1601/nm.4999 were renamed [25]. Shortly after, 10.1601/nm.4999 and numerous other 10.1601/nm.4857 species were reclassified as 10.1601/nm.5109 spp. [27]. Both 10.1601/nm.5116 and 10.1601/nm.5156 share the hallmarks of other 10.1601/nm.5109 species in that they are facultative anaerobes, that grow well on nutrient agar at neutral pH but inclusion of a fermentable sugar, such as glucose, will enhance growth [28]. These paenibacilli produce similar colonies when grown for 24 h on tryptic soy agar and appear circular, entire, and translucent, but are distinguishable by yellow pigmentation of 10.1601/nm.5116 colonies, which is not seen with 10.1601/nm.5156. Both 10.1601/nm.5116 and 10.1601/nm.5156 can respire anaerobically using nitrate as an electron acceptor. Both can break down disaccharides and some polysaccharides [27]. Carbon sources that support growth and complex organic compounds that 10.1601/nm.5116 and 10.1601/nm.5156 can hydrolyze are listed in Tables 1 and 2, respectively. Unlike 10.1601/nm.5116, 10.1601/nm.5156 can ferment lactose as well as the sugar alcohols D-mannitol and D-sorbitol [24, 25]. Another distinguishing characteristic is the ability of 10.1601/nm.5156 to produce indole. The ability to decompose thiamin was considered a distinct feature of 10.1601/nm.5156 [24] but can no longer be used to differentiate it from 10.1601/nm.5116 or 10.1601/nm.5127 (unpublished). 10.1601/nm.5116 is closely related to the honeybee pathogen 10.1601/nm.5114, while 10.1601/nm.5156 is very closely related to 10.1601/nm.5127, 10.1601/nm.5152, and 10.1601/nm.5144, the latter two species are insect pathogens, responsible for milky spore disease in Japanese beetles [29]. Recently it was discovered that paenibacilli are distinct from 10.1601/nm.4857 spp. in the arrangement of genes around the chromosomal origin of replication [30]. Paenibacilli code for a YheC/D family protein, designated *orf14*, between the *gyrA* and *gyrB* genes while 10.1601/nm.4857 species do not have this intervening gene. Our maximum likelihood 16S rRNA gene tree generated by FastTree 2.1 [31] is congruent with these studies (Fig. 2). The tree also indicates that 10.1601/nm.5156 OSY-SE is a strain of 10.1601/nm.5116.

The present study was used to learn more about the genomic context of the thiaminase I gene and thiamin metabolism in these paenibacilli and their close relative 10.1601/nm.5127 C454 which has a published draft genome [32].

## Genome sequencing information

### Genome project history

Both 10.1601/nm.5156 10.1601/strainfinder?urlappend=%3Fid%3DNRRL+B-4156 and 10.1601/nm.5116 10.1601/strainfinder?urlappend=%3Fid%3DNRRL+B-23460 were acquired from the Agricultural Research Service Culture Collection. The DNA was sequenced in April of 2014. Raw reads were assembled using SPAdes version 3.5 [33]. The contigs were quality filtered by size and coverage. Completeness and heterogeneity were assessed using CheckM [34] and the draft genomes were submitted to Genoscope for annotation with the MicroScope platform [35]. The assembled draft genomes were submitted to the Joint Genome Institute Integrated Microbial Genomes analysis system [36] in October 2016 for annotation. Project summaries are provided in Table 3.

**Table 3:** Project information

MIGS ID	Property	Term (10.1601/nm.5116)	Term (10.1601/nm.5156)
MIGS 31	Finishing quality	Draft	Draft
MIGS-28	Libraries used	TruSeq	TruSeq
MIGS 29	Sequencing platforms	Illumina MiSeq	Illumina MiSeq
MIGS 31.2	Fold coverage	150×	150×
MIGS 30	Assemblers	10.1601/strainfinder?urlappend=%3Fid%3DSPA+des+5.3	SPAdes 3.5
MIGS 32	Gene calling method	IMG and MicroScope	IMG and MicroScope
	Locus Tag	Ga0138518	Ga0138519
	Genbank ID	NDGJ00000000	NDGK00000000
	GenBank Date of Release	05/31/2017	05/31/17
	GOLD ID	Ga0138518	Ga0138519
	BIOPROJECT	PRJNA382554	PRJNA382555
MIGS 13	Source Material Identifier	Insect associated	Human associated
	Project relevance	Metabolic pathways	Metabolic pathways

### Growth conditions and genomic DNA preparation

Both 10.1601/nm.5116 10.1601/strainfinder?urlappend=%3Fid%3DNRRL+B-23460 and 10.1601/nm.5156 10.1601/strainfinder?urlappend=%3Fid%3DNRRL+B-4156 were grown in tryptic soy broth with shaking, at 30 °C and 37 °C, respectively. Genomic DNA was extracted using a protocol typically used for isolating high molecular weight DNA from 10.1601/nm.10618 [37]. Briefly, cells were lysed with lysozyme and sodium n-lauryl sarcosine. DNA was extracted using phenol:chloroform, and precipitated using ethanol. Near-complete 16S rRNA genes were amplified from the genomic DNA. Sequences were determined and compared with published sequences available in GenBank. The whole genome sequencing projects for 10.1601/nm.5116 10.1601/strainfinder?urlappend=%3Fid%3DNRRL+B-23460 and 10.1601/nm.5156 10.1601/strainfinder?urlappend=%3Fid%3DNRRL+B-4156 were deposited in DDBJ/EMBL/GenBank under accession numbers NDGJ00000000 and NDGK00000000, respectively.

### Genome sequencing and assembly

Illumina MiSeq 2 × 250 sequencing reactions were conducted on the two DNA samples at the Cornell University Institute of Biotechnology in Ithaca, NY. This resulted in 3,704,766 reads for the 10.1601/nm.5116 10.1601/strainfinder?urlappend=%3Fid%3DNRRL+B-23460 genome and 4,092,728 reads for the *P. thiamonolyticus* 10.1601/strainfinder?urlappend=%3Fid%3DNRRL+B-4156 genome. The reads were quality checked and assembled using 10.1601/strainfinder?urlappend=%3Fid%3DSPA+des+3.5 [33]. Contigs were filtered based on coverage (above 50×) and size (above 1000 bp). CheckM [34] was used to determine genome completeness and revealed that the 10.1601/nm.5116 10.1601/strainfinder?urlappend=%3Fid%3DNRRL+B-23460 genome is 99.73% complete with no strain heterogeneity, while the 10.1601/nm.5156 genome is 99.68% complete with no strain heterogeneity.

### Genome annotation

Gene calling and annotations for 10.1601/nm.5116 10.1601/strainfinder?urlappend=%3Fid%3DNRRL+B-23460 and 10.1601/nm.5156 10.1601/strainfinder?urlappend=%3Fid%3DNRRL+B-4156 were developed by both the MicroScope platform [35] and IMG [36], 10.1601/nm.5127 C454 was annotated with MicroScope only. Annotations of interest were independently verified using the Uniprot (Swissprot and TrEMBL) database and BLAST. Ambiguous gene sequences were compared to their 10.1601/nm.10618 counterparts to further help identify a putative function. DELTA-BLAST was used to determine functional domains of uncharacterized proteins, and confirm those of characterized proteins of interest.

## Genome properties

The draft genome for 10.1601/nm.5116 10.1601/strainfinder?urlappend=%3Fid%3DNRRL+B-23460 is 5,404,821 bp (50.49% G + C) and comprises 51 contigs. The largest contig is 827,142 bp, and the smallest is 1010 bp in length. The N50 of the genome is 280,248. IMG identified 4957 genes in the genome. Of those genes, 4822 encode for proteins (97.28%), 22 are rRNA genes (0.44%), 76 are tRNA genes (1.53%), and no pseudogenes were discovered. Of the 22 rRNA genes identified, seven are 5S, ten are 16S, and five are 23S genes. The draft genome for 10.1601/nm.5156 is 6,547,709 bp (53.64% G + C), contains 48 contigs, with the largest contig being 1,172,336 bp and the smallest being 1148 bp. The N50 is 254,830 bp. For this genome 5880 genes were identified as protein encoding (97.89%), with 21 rRNA genes (0.36%), 77 tRNA genes (1.31%), and no pseudogenes (0.00%). Amongst the rRNA genes, five 5S, nine 16S, and seven 23S genes were identified. More details of these draft genomes are given in Table 4, and the CoG analyses are summarized in Tables 5 and 6.

**Table 4:** Genome and annotation statistics for 10.1601/nm.5116 and 10.1601/nm.5156

Attribute	10.1601/nm.5116 NRRL B-23460		10.1601/nm.5156 10.1601/strainfinder?urlappend=%3Fid%3DNRRL+B-4156	
	Value	% of Total	Value	% of Total
Genome size (bp)	5,404,821	100.00	6,537,496	100.00
DNA coding (bp)	4,642,405	85.89	5,508,364	84.26
DNA G + C (bp)	2,729,114	50.49	3,507,168	53.65
DNA scaffolds	51	100.00	47	100.00
Total genes	4957	100.00	5880	100.00
Protein coding genes	4822	97.28	5756	97.89
RNA genes	135	2.72	124	2.11
Pseudo genes	0	0	0	0
Genes in internal clusters	1259	25.40	1709	29.06
Genes with function prediction	3756	75.77	4458	75.82
Genes assigned to COGs	3092	62.38	3654	62.14
Genes with Pfam domains	3910	78.88	4674	79.49
Genes with signal peptides	304	6.13	350	5.95
Genes with transmembrane helices	1385	27.94	1658	28.20
CRISPR repeats	0	0	0	0

**Table 5:** Number of genes associated with general COG functional categories

Code	Value	% of total	Description
J	219	6.30%	Translation, ribosomal structure and biogenesis
A	0	0.00%	RNA processing and modification
K	343	9.87%	Transcription
L	98	2.82%	Replication, recombination and repair
B	1	0.03%	Chromatin structure and dynamics
D	50	1.44%	Cell cycle control, Cell division, chromosome partitioning
V	110	3.16%	Defense mechanisms
T	194	5.58%	Signal transduction mechanisms
M	179	5.15%	Cell wall/membrane biogenesis
N	66	1.90%	Cell motility
U	29	0.83%	Intracellular trafficking and secretion
O	112	3.22%	Posttranslational modification, protein turnover, chaperones
C	165	4.75%	Energy production and conversion
G	368	10.59%	Carbohydrate transport and metabolism
E	317	9.12%	Amino acid transport and metabolism
F	103	2.96%	Nucleotide transport and metabolism
H	186	5.35%	Coenzyme transport and metabolism
I	127	3.65%	Lipid transport and metabolism
P	213	6.13%	Inorganic ion transport and metabolism
Q	102	2.93%	Secondary metabolites biosynthesis, transport and catabolism
R	281	8.08%	General function prediction only
S	186	5.35%	Function unknown
–	1865	37.62%	Not in COGs

The total is based on the total number of protein coding genes in the genome of 10.1601/nm.5116 10.1601/strainfinder?urlappend=%3Fid%3DNRRL+B-23460

**Table 6:** Number of genes associated with general COG functional categories

	Value	% of total	Description
J	248	6.03%	Translation, ribosomal structure and biogenesis
A	0	0.00%	RNA processing and modification
K	431	10.47%	Transcription
L	112	2.72%	Replication, recombination and repair
B	0	0.00%	Chromatin structure and dynamics
D	59	1.43%	Cell cycle control, Cell division, chromosome partitioning
V	157	3.81%	Defense mechanisms
T	263	6.39%	Signal transduction mechanisms
M	224	5.44%	Cell wall/membrane biogenesis
N	63	1.53%	Cell motility
U	28	0.68%	Intracellular trafficking and secretion
O	142	3.45%	Posttranslational modification, protein turnover, chaperones
C	199	4.83%	Energy production and conversion
G	450	10.93%	Carbohydrate transport and metabolism
E	370	8.99%	Amino acid transport and metabolism
F	109	2.65%	Nucleotide transport and metabolism
H	195	4.74%	Coenzyme transport and metabolism
I	139	3.38%	Lipid transport and metabolism
P	246	5.98%	Inorganic ion transport and metabolism
Q	118	2.87%	Secondary metabolites biosynthesis, transport and catabolism
R	334	8.11%	General function prediction only
S	202	4.91%	Function unknown
–	2226	37.86%	Not in COGs

The total is based on the total number of protein coding genes in the genome of 10.1601/nm.5156 10.1601/strainfinder?urlappend=%3Fid%3DNRRL+B-4156

## Insights from the genome sequence

We investigated the potential thiamin biosynthetic capabilities of 10.1601/nm.5116 10.1601/strainfinder?urlappend=%3Fid%3DNRRL+B-23460, 10.1601/nm.5156 10.1601/strainfinder?urlappend=%3Fid%3DNRRL+B-4156, and 10.1601/nm.5127 C454 using the annotations and metabolic pathways generated by MicroScope. Typically in bacteria, TPP, the active cofactor, is formed from two phosphorylated moieties, THZ-P and HMP-PP. The thiazole moiety is derived from the glycolysis products pyruvate and G3P, a sulfur from cysteine, and either tyrosine (in *E. coli*) or glycine (in 10.1601/nm.10618) [38]. The formation of THZ-P requires a suite of proteins including deoxy-d-xylulose 5-phosphate synthase (Dxs), a sulfur donor protein (NifS or IscS), adenyltransferase (ThiF), sulfur carrier protein (ThiS), thiazole synthase (ThiG), thiazole biosynthesis protein ThiH or glycine oxidase ThiO, and in some cases an aromatase (TenI) [38]. The pyrimidine moiety is derived from AIR, an intermediate in purine biosynthesis. HMP synthase (ThiC) and HMP kinase (ThiD) are required to form HMP-PP [38]. Thiamin phosphate synthase (ThiE) combines THZ-P and HMP-PP to form TMP, which is then phosphorylated by thiamin phosphate kinase (ThiL), forming TPP [38].

**Fig. 3:**
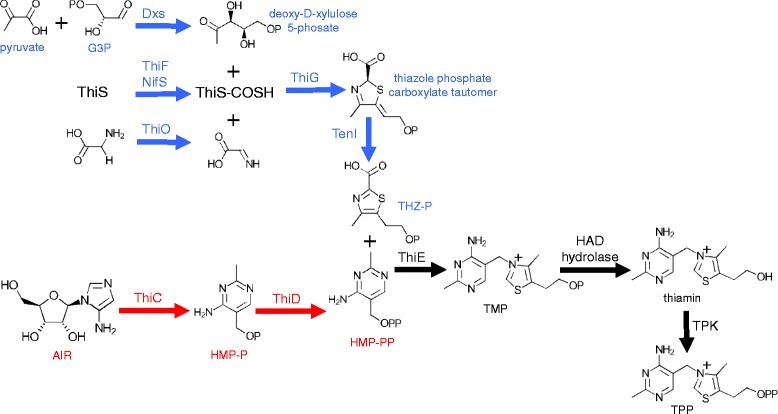
Predicted thiamin biosynthesis pathway in 10.1601/nm.5116 10.1601/strainfinder?urlappend=%3Fid%3DNRRL+B-23460. Pathways involved in thiazole biosynthesis are highlighted in blue and pathways involved in pyrimidine biosynthesis are shown in red. The steps in black correspond to the coupling of the thiazole and pyrimidine moieties, and the formation of the active cofactor TPP

**Fig. 4:**
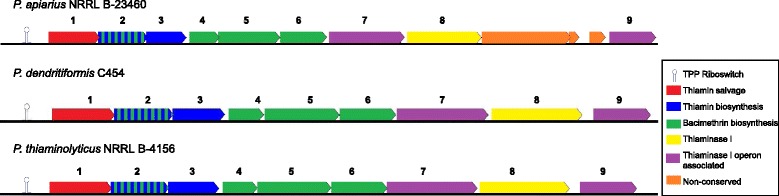
Putative thiaminase I operons in the three 10.1601/nm.5109 species. Annotations of conserved genes shared amongst the three species are as follows (1) *thiM;* (2) *thiD*; (3) *thiE*; (4) glycosyltransferase; (5) thymidylate synthase; (6) methyltransferase; (7) *yzgD* NUDIX hydrolase; (8) thiaminase I and (9) HAD hydrolase. Three genes in 10.1601/nm.5116 B23460 that are not conserved include a putative transcriptional regulator and two genes of unknown function. The operons may be under the control of a putative thiamin pyrophosphate binding riboswitch, upstream of *thiM*. Genes are color-coded based on proposed functions

The 10.1601/nm.5116 draft genome contains all of the genes required to make THZ-P (Fig. 3); *thiG*, *thiO*, *thiS*, and *tenI* are located in an operon putatively regulated by a TPP-binding riboswitch. The presence of *thiO* suggests that 10.1601/strainfinder?urlappend=%3Fid%3DNRRL+B-23460 uses glycine to generate the thiazole moiety. With *thiC* and *thiD* present, it appears competent for HMP-PP biosynthesis (3). The genome contains *thiE* but lacks *thiL* for the terminal phosphorylation; however, it contains a putative TPK. Plants, fungi, and a few species of bacteria, use a different thiamin biosynthesis strategy where thiamin monophosphate is dephosphorylated to thiamin, and then converted to TPP by a TPK [39]. The genome content of 10.1601/nm.5116 10.1601/strainfinder?urlappend=%3Fid%3DNRRL+B-23460 suggests that it synthesizes thiamin in this manner. Hasnain and colleagues recently demonstrated that HAD-superfamily enzymes of the subfamily IA in plants and some bacteria catalyze the dephosphorylation of TMP [39]. In bacteria, these hydrolase genes are either fused to a thiamin biosynthesis gene, like *thiD* or *thiE*, or these genes are located in the same operon. The 10.1601/nm.5116 10.1601/strainfinder?urlappend=%3Fid%3DNRRL+B-23460 genome has a HAD subfamily IA hydrolase gene that potentially serves this function, and is located in an operon with other thiamin biosynthesis genes (Fig. 4).

**Fig. 5:**
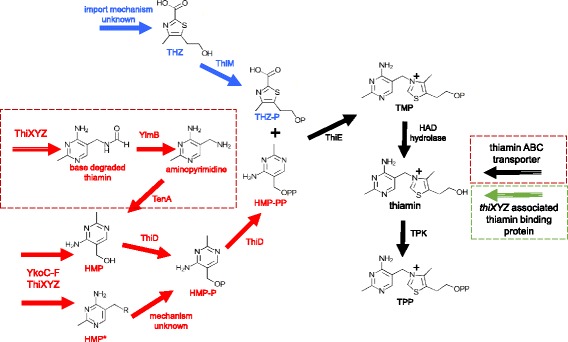
Predicted thiamin salvage pathways in all three paenibacilli. Pathways involved in thiazole salvage are highlighted in blue and pathways involved in pyrimidine salvage are shown in red. The dotted red boxes are steps unique to 10.1601/nm.5156 10.1601/strainfinder?urlappend=%3Fid%3DNRRL+B-4156 and 10.1601/nm.5127 C454. Biosynthetic pathways are shown with solid arrows and import pathways are indicated with a striped arrow. The putative importer boxed in green is unique to 10.1601/nm.5116 10.1601/strainfinder?urlappend=%3Fid%3DNRRL+B-23460. In all cases, it is not yet understood how THZ enters the cell

The genes *thiD*, *thiE*, and HAD subfamily IA hydrolase gene are found in a highly conserved operon with the thiaminase I gene in the 10.1601/nm.5116 10.1601/strainfinder?urlappend=%3Fid%3DNRRL+B-23460, 10.1601/nm.5156 10.1601/strainfinder?urlappend=%3Fid%3DNRRL+B-4156 and 10.1601/nm.5127 C454 draft genomes. The operons of all three strains are depicted in Fig. 4 and appear to be regulated by a TPP-binding riboswitch [40]. Thiazole kinase (*thiM*), which phosphorylates THZ (Fig. 5) [38] is the first gene in the operon, followed by *thiD* and *thiE.* The thiamin biosynthesis genes are proceeded by a nucleoside 2-deoxyribosyltransferase, a thymidylate synthase, a SAM-dependent methyltransferase, a Nudix-family hydrolase (YzgD), and thiaminase I (Fig. 4). In the 10.1601/nm.5156 10.1601/strainfinder?urlappend=%3Fid%3DNRRL+B-4156 and 10.1601/nm.5127 C454 operons, a HAD subfamily IA hydrolase is located directly after the thiaminase I gene. In 10.1601/nm.5116 10.1601/strainfinder?urlappend=%3Fid%3DNRRL+B-23460, three additional genes are present, which code for a putative transcriptional regulator and two proteins of unknown function (Fig. 4). Since the HAD hydrolase is in the same operon as *thiD* and *thiE*, it is likely that it performs the dephosphorylation of TMP. Biochemical studies by Tirrell and colleagues reveal that the YzgD Nudix hydrolase has a HAD domain, which specifically cleaves pyridoxal phosphate, but does not dephosphorylate TMP, TPP, or THZ-P although HMP-P was not tested [41]. The Nudix hydrolase domain is more promiscuous as it is active on nucleoside diphosphates such as CDP-alcohols, ADP-coenzymes, ADP-ribose, TDP-glucose, and some UDP-sugars, restoring the nucleoside monophosphate [41]. It is unclear how this enzyme relates to thiaminase I, but it may play a role in thiamin metabolism. Recently, Nudix hydrolases were discovered clustered with thiamin biosynthesis genes in a few bacterial species as well as in plants and yeast. These Nudix proteins are able to hydrolyze a phosphate from the diphosphate forms of oxothiamin and oxythiamin, thiamin oxidation and hydrolysis products respectively, providing these cells with resistance to these toxic analogs [42]. Due to its location in the paenibacilli genomes, it may serving this protective function, preventing the cell from using toxic thiamin analogs as cofactors instead of TPP.

Cooper et al. described the bacimethrin operon of 10.1601/nm.3901, which includes the thiaminase I gene [43]. Bacimethrin is a toxic analog of HMP, that when combined with THZ-P forms the antivitamin 2′-methoxythiamin pyrophosphate, which binds enzymes in place of the TPP cofactor, thus rendering the enzyme nonfunctional [43–45]. The bacimethrin operon consists of a glycosyltransferase (nucleoside 2-deoxyribosyltransferase), thymidylate synthase, methyltransferase, thiaminase I, and pyrimidine kinase, all of which are present in the three paenibacilli (Fig. 4), making it likely that they can produce bacimethrin [43]. In the paenibacilli, ThiD may be bifunctional, serving as a kinase for both pyrimidines, phosphorylating bacimethrin as well as HMP-P. The function of thiaminase I when the antivitamin is produced is not known. Since thiaminase I does not degrade 2′-methoxythiamin pyrophosphate in 10.1601/nm.3901 [43], it is possible that thiaminase I could enhance the effectiveness of this antibiotic against competing bacteria*.* In contrast to the paenibacilli operons, the 10.1601/nm.3901 thiaminase I operon does not contain genes involved in thiamin biosynthesis and salvage. The 10.1601/nm.3901 operon also contains a putative ABC transporter that is not found in the paenibacilli thiaminase I operons.

Apparently, 10.1601/nm.5127 C454 and 10.1601/nm.5156 10.1601/strainfinder?urlappend=%3Fid%3DNRRL+B-4156 lack the genomic potential to synthesize both moieties of thiamin. Of the genes involved in thiazole biosynthesis, they both have *dxs* and *nifS*, and 10.1601/strainfinder?urlappend=%3Fid%3DNRRL+B-4156 contains *thiF*. Neither has *thiO* which is essential for thiazole synthesis in 10.1601/nm.10618. Both lack *thiC*, so they are unable to convert AIR to HMP. The presence of *thiD* and *thiE* in their thiaminase I operons provides the potential to make TMP from environmentally acquired THZ and HMP, a strategy used by other bacteria [46]. ThiM can phosphorylate environmentally derived thiazole alcohol, which can be combined with HMP-P by ThiE (Fig. 5). Like 10.1601/nm.5116 10.1601/strainfinder?urlappend=%3Fid%3DNRRL+B-23460, their genomes encode TPK to make TPP. The presence of the thiaminase I in the same operon as *thiM*, *thiD*, and *thiE* suggests a potential role in thiamin salvage. The thiaminase I, acting on thiamin or pyrithiamine (a thiamin analog) [10] would generate HMP* and a free THZ. We propose that THZ and HMP* could be imported into the cell, phosphorylated by their respective kinases, and combined by ThiE (Fig. 5). TPP is then produced via dephosphorylation by the HAD hydrolase and addition of the pyrophosphate by TPK.

The potential pathways available to the three paenibacilli to salvage thiamin are summarized in Fig. 5. Both 10.1601/nm.5127 C454 and 10.1601/nm.5156 10.1601/strainfinder?urlappend=%3Fid%3DNRRL+B-4156 genomes code for the intracellular enzyme thiaminase II (TenA), but 10.1601/nm.5116 10.1601/strainfinder?urlappend=%3Fid%3DNRRL+B-23460 lacks this gene. Thiaminase II catalyzes the base exchange of thiamin with water, but is not a thiaminase I homolog [47]. It functions in the salvage of HMP from base-degraded thiamin [47]. In both genomes that code for this enzyme, TenA appears regulated by a TPP riboswitch. The genomes of all three paenibacilli contain *ylmB*, which deacetylates base-degraded thiamin forming aminopyrimidine, the preferred substrate for TenA [47] (Fig. 5).

MicroScope identified another TPP riboswitch in all three genomes that appears to regulate a transport system. In all three operons, the riboswitch is preceded by an NMT1/Thi5 domain protein. Thi5 is a yeast protein that converts pyridoxal and histidine to HMP-P, and is a homolog to the ThiY protein found in 10.1601/nm.4885 and 10.1601/nm.4919 [48]. ThiY is part of the ThiXYZ ABC transport system putatively involved in the uptake of HMP, as well as in the uptake of base-degraded thiamin [47–49]. In 10.1601/nm.5116 10.1601/strainfinder?urlappend=%3Fid%3DNRRL+B-23460, this ThiY homolog is followed by a small, 98 amino acid protein with a thiamin-binding domain, suggesting it may have two alternative transporters for this system. However, in the other two paenibacilli genomes, this is followed by a permease, and the ATP-binding protein of the ABC transport system. The other two ABC transport proteins are found after the small thiamin-binding protein in 10.1601/strainfinder?urlappend=%3Fid%3DNRRL+B-23460. This suggests that the genomes of all three paenibacilli contain the ThiXYZ HMP transport system, or a homologous system. In 10.1601/nm.5156 10.1601/strainfinder?urlappend=%3Fid%3DNRRL+B-4156 and 10.1601/nm.5127 C454, the system could potentially be dedicated for base-degraded thiamin uptake. The lack of TenA in 10.1601/nm.5116 10.1601/strainfinder?urlappend=%3Fid%3DNRRL+B-23460 may explain why it has an additional thiamin-binding protein associated with this transport system, as it cannot use base-degraded thiamin. It is also plausible that this system is involved in the uptake of the HMP* generated by thiaminase I in all three species.

All three paenibacilli contain the *ykoC*-*F* operon, which encodes for a putative ABC transport system for HMP uptake [50]. The genes encode for two transmembrane components, an ATPase, and an HMP/thiamin-binding protein YkoF [50]. It is unclear if this system takes up both HMP and thiamin, or is specific for HMP and HMP derivatives, as YkoF binds the HMP moiety and does not appear to have any residues to anchor the thiazole moiety of thiamin. This is in contrast to thiamin binding by TbpA, which also binds the THZ [12]. The YkoF transporter could potentially be used for the uptake of the HMP* derived from thiaminase I breakdown of thiamin and thiamin analogs, as well as free HMP, and possibly base-degraded thiamin as well. MicroScope identified a TPP-binding riboswitch upstream of this operon in all three paenibacilli genomes, suggesting that its expression is regulated by thiamin availability.

The 10.1601/nm.5127 C454 and 10.1601/nm.5156 10.1601/strainfinder?urlappend=%3Fid%3DNRRL+B-4156 genomes contain another thiamin ABC transport permease in addition to the YkoC-F system. The thiamin permeases in these two genomes appear to be regulated by TPP riboswitchs and share amino acid sequence similarity with YkoD. Next to the permease is the ATP-binding protein, and the third gene in the operon encodes another transmembrane permease with homology to the cobalt ABC transporter permease CbiQ. The presence of this permease suggests that the *yko* system is only used in HMP and HMP derivative uptake and this system is specific for thiamin, allowing for the two thiamin auxotrophs to acquire intact thiamin from the environment. In all three genomes, ThiW, a transporter specific for THZ [51, 52], was not identified. It is possibly they acquire environmental THZ via an unknown mechanism.

## Conclusions

The genome sequences of 10.1601/nm.5116 10.1601/strainfinder?urlappend=%3Fid%3DNRRL+B-23460 and 10.1601/nm.5156 10.1601/strainfinder?urlappend=%3Fid%3DNRRL+B-4156 reveal insights into thiamin metabolism of these organisms. While 10.1601/nm.5116 10.1601/strainfinder?urlappend=%3Fid%3DNRRL+B-23460 appears capable of synthesizing thiamin *de novo*, 10.1601/nm.5156 10.1601/strainfinder?urlappend=%3Fid%3DNRRL+B-4156 is not, as it lacks the ability to make HMP and THZ. Both organisms apparently phosphorylate thiamin to its active form in a manner rarely used in bacteria, as they can dephosphorylate TMP and then add two phosphates with a pyrophosphokinase to make TPP. The thiaminase I gene is located in a putatively TPP riboswitch-regulated operon with genes for the synthesis of bacimethrin, as well as thiamin biosynthesis and salvage genes. This suggests a potential metabolic role for thiaminase I in thiamin synthesis, especially in 10.1601/nm.5156 10.1601/strainfinder?urlappend=%3Fid%3DNRRL+B-4156, which cannot synthesize thiamin precursors. Further, both species appear to have two different systems to take up HMP, both of which appear to be regulated with TPP riboswitches. It is possible that one of these transport systems is specific for HMP* generated from thiaminase I. We suggest that this HMP* compound can be used in thiamin biosynthesis along with THZ scavenged from the breakdown of thiamin. 10.1601/nm.5156 10.1601/strainfinder?urlappend=%3Fid%3DNRRL+B-4156 has the ability to salvage base-degraded thiamin with its intracellular thiaminase II [47], whereas 10.1601/nm.5116 10.1601/strainfinder?urlappend=%3Fid%3DNRRL+B-23460 does not. This is another method in which 10.1601/nm.5156 can acquire the pyrimidine precursor for thiamin. To further compensate for its auxotrophy, 10.1601/nm.5156 10.1601/strainfinder?urlappend=%3Fid%3DNRRL+B-4156 may have a thiamin specific ABC transport system, which is not present in the 10.1601/nm.5116 10.1601/strainfinder?urlappend=%3Fid%3DNRRL+B-23460 genome. However, 10.1601/strainfinder?urlappend=%3Fid%3DNRRL+B-23460 has a unique thiamin-binding protein encoded for in the *thiXYZ* operon which 10.1601/nm.5156 10.1601/strainfinder?urlappend=%3Fid%3DNRRL+B-4156 lacks. Biochemical and genetic tests need to be conducted to test the hypotheses generated in this study to further elucidate the roles these genes and proteins play in thiamin metabolism.

## Abbreviations

AIR: phospho5-aminoimidazole ribotide
G3P: Glyceraldehyde-3-phosphate
HMP: hydroxymethyl pyrimidine
HMP*: hydroxymethyl pyrimidine-organic nucleophile
HMP-P: hydroxymethyl pyrimidine phosphate
HMP-PP: hydroxymethyl pyrimidine pyrophosphate
THZ: thiazole carboxylate
THZ-P: thiazole phosphate carboxylate
TMP: thiamin monophosphate
TPK: thiamin pyrophosphokinase
TPP: thiamin pyrophosphate
